# Empowering Smart Aging: Insights into the Technical Architecture of the e-VITA Virtual Coaching System for Older Adults

**DOI:** 10.3390/s24020638

**Published:** 2024-01-19

**Authors:** Riccardo Naccarelli, Francesca D’Agresti, Sonja Dana Roelen, Kristiina Jokinen, Sara Casaccia, Gian Marco Revel, Martino Maggio, Zohre Azimi, Mirza Mohtashim Alam, Qasid Saleem, Abrar Hyder Mohammed, Giulio Napolitano, Florian Szczepaniak, Mossaab Hariz, Gérard Chollet, Christophe Lohr, Jérôme Boudy, Rainer Wieching, Toshimi Ogawa

**Affiliations:** 1 Department of Industrial Engineering and Mathematical Sciences, Polytechnic University of Marche, 60131 Ancona, Italy; s.casaccia@univpm.it (S.C.); gm.revel@univpm.it (G.M.R.); 2 Engineering Ingegneria Informatica SpA, 00144 Roma, Italy; francesca.dagresti@eng.it (F.D.); martino.maggio@eng.it (M.M.); 3 Institut für Experimentelle Psychophysiologie GmbH, 40215 Düsseldorf, Germany; s.roelen@ixp-duesseldorf.de (S.D.R.); z.azimi@ixp-duesseldorf.de (Z.A.); 4 Artificial Intelligence Research Center, National Institute of Advanced Industrial Science and Technology (AIRC/AIST), Tokyo 135-0064, Japan; kristiina.jokinen@aist.go.jp; 5 Leibniz Institute for Information Infrastructure, FIZ Karlsruhe, 76344 Eggenstein-Leopoldshafen, Germany; mirza-mohtashim.alam@fiz-karlsruhe.de; 6 Institute for Applied Informatics (InfAI), 04109 Leipzig, Germany; qasid.saleem@infai.org (Q.S.); hyder@infai.org (A.H.M.); napolitano@infai.org (G.N.); 7 Institut Mines-Télécom (IMT), 91120 Palaiseau, France; florian.szczepaniak@telecom-sudparis.eu (F.S.); mossaab.hariz@telecom-sudparis.eu (M.H.); gerard.chollet@telecom-sudparis.eu (G.C.); christophe.lohr@imt-atlantique.fr (C.L.); jerome.boudy@telecom-sudparis.eu (J.B.); 8 Institute for Business Informatics & New Media, University Siegen, Kohlbettstr. 15, 57072 Siegen, Germany; rainer.wieching@uni-siegen.de; 9 Smart-Aging Research Center, Tohoku University, Sendai 980-8575, Japan; toshimi.ogawa.e6@tohoku.ac.jp

**Keywords:** smart aging, technical architecture, sensors, virtual coach, active and healthy aging, older adults

## Abstract

With a substantial rise in life expectancy throughout the last century, society faces the imperative of seeking inventive approaches to foster active aging and provide adequate aging care. The e-VITA initiative, jointly funded by the European Union and Japan, centers on an advanced virtual coaching methodology designed to target essential aspects of promoting active and healthy aging. This paper describes the technical framework underlying the e-VITA virtual coaching system platform and presents preliminary feedback on its use. At its core is the e-VITA Manager, a pivotal component responsible for harmonizing the seamless integration of various specialized devices and modules. These modules include the Dialogue Manager, Data Fusion, and Emotional Detection, each making distinct contributions to enhance the platform’s functionalities. The platform’s design incorporates a multitude of devices and software components from Europe and Japan, each built upon diverse technologies and standards. This versatile platform facilitates communication and seamless integration among smart devices such as sensors and robots while efficiently managing data to provide comprehensive coaching functionalities.

## 1. Introduction

Due to a significant rise in life expectancy over the last century, societies are now faced with the complex task of addressing the challenges presented by a growing, aging population. Consequently, there is an urgent need to prioritize the development of smart living solutions tailored to the care of older individuals and the promotion of active and healthy aging [1,2,3]. To meet this need, an innovative virtual coaching system was developed as part of a collaborative research initiative between Europe (H2020) and Japan (MIC), spanning a duration of three years [4]. The primary objective of the e-VITA virtual coach is to enable older individuals to enhance their ability to oversee their overall well-being and daily routines. This virtual coach provides guidance and motivation across essential dimensions of active and healthy aging, covering cognition, physical activity, mobility, emotional well-being, social engagement, leisure activities, and spirituality. This approach aims to improve overall well-being and engagement among stakeholders. By leveraging big data analytics, social-emotional computing, and unobtrusive sensor technology, the virtual coach offers individualized profiling and custom-tailored recommendations. It identifies potential risks within the user’s living environment and provides assistance through natural interactions using various technologies such as 3D-pseudo holograms (Pepper Ghost), emotional objects, and robotic systems. This incorporates multimodal and spoken dialogue technology, as well as advanced knowledge graphs and knowledge base research [5]. 

The initial phase of the e-VITA project involved studies and technical analysis that highlighted the complex and diverse nature of the e-VITA technological assets, which comprise various devices and software components employing different technologies and standards across Europe and Japan. The project faced the challenge of establishing a cohesive technical architecture due to this diversity. Different project work packages addressed various aspects of this complexity, including sensing devices, data harmonization, semantics, and dialog systems. The purpose of this paper is to provide a comprehensive description of the e-VITA platform architecture, which incorporates technical assets from different project partners, while also presenting preliminary feedback from older users on its use in real-world scenarios. This architecture serves as the foundation for the e-VITA coaching system, facilitating communication and integration among smart devices, data harmonization, processing, analytics, security, and privacy measures in compliance with legal frameworks like the General Data Protection Regulation (GDPR) and the Japanese Act on Protection of Personal Information (APPI). 

The paper outlines the functionalities, data exchange processes, and relationships among components. The foundational framework and baseline platform for seamless integration is the Digital Enabler (DE), an ecosystem platform crafted by Engineering Ingegneria Informatica SpA. This platform has been used to integrate the devices and modules and to provide the main data capabilities. It relies primarily on open components, with a significant emphasis on utilizing elements from the FIWARE framework [6], extended and customized to fit project requirements. The DE is designed for collecting, analyzing, and presenting dispersed data from various sources, facilitating multi-domain data integration, harmonization, and interoperability across multiple devices. The DE is built as a modular, micro-services-oriented, and containerized platform, incorporating open-source and custom-developed elements. The platform’s components communicate through standardized APIs like ETSI’s NGSI-LD. DE exploits the Context Broker and Device Manager (DEMA) to manage context information from devices and sensors. The DEMA, powered by FIWARE’s Internet of Things (IoT) Agents, connects IoT devices to the platform. The Data Mashup component enables harmonization and data integration by visually defining data pipelines. DE’s capabilities also encompass Cloudev for developing, running, and managing application functionalities using the Function as a Service (FaaS) model. Authentication, authorization, and personal data management are handled through Keycloak [7].

Drawing inspiration from prior research projects, the e-VITA initiative thus undertook the task of developing an advanced virtual coach. It capitalizes on the newly emerged capabilities of OpenAI’s ChatGPT to assist older users from diverse cultural backgrounds in their daily lives. This is achieved through the creation of a platform that seamlessly integrates diverse data from sensors, wearables, robots, and smart devices.

In summary, this paper provides a focused exploration of the e-VITA project’s advancements in virtual coaching systems. It highlights innovations in the technical architecture and the development of an advanced virtual coach, emphasizing the integration of cutting-edge technologies, open-source components, and a user-centric approach. Notable features include the use of smart environmental sensing for real-time contextual dialogue tailoring and the incorporation of advanced elements such as multimodal, multidomain, and multilingual components facilitated by OpenAI’s ChatGPT-3.5 Turbo. The paper also underscores e-VITA’s emphasis on a multicultural context, ensuring inclusivity and relevance for users from diverse cultural backgrounds. These aspects collectively position e-VITA as a significant undertaking in the domain of virtual coaching for the active and healthy aging of community-dwelling older adults in smart environments.

The paper is organized as follows: Section 2 gives an overview of the most recent related state-of-the-art work, while Section 3 presents the architecture of the e-VITA platform. Section 4 describes all the components employed within the e-VITA platform, encompassing sensor-based multimodal Data Fusion, emotion recognition, conversational artificial intelligence (AI), visualization, and end-user applications. Finally, after presenting preliminary feedback from older users on the e-VITA platform test in Section 5, Section 6 concludes the paper.

## 2. Related Work

In the dynamic landscape of healthcare, the intersection of technology and aging has spurred innovative solutions to enhance the well-being and quality of life of older adults. E-health platforms, specifically tailored for smart aging, represent a promising avenue for addressing the unique health challenges associated with aging populations. Over the past decade, significant attention has been directed toward exploring the potential of these platforms to promote health, prevent disease, and facilitate active aging, particularly in the context of conversational agents (CAs) [8]. Given that language serves as the primary means of establishing human connections and considering the expanding capabilities of voice services and natural language understanding, CAs emerge as compelling intervention interfaces for virtual coaching. Virtual coaches, defined as systems able to perceive relevant context, determine user intent, and provide constructive feedback to improve some aspect of the user’s life, leverage various interfaces of CAs incorporating distinct interaction modalities like images, speech, and text. The diverse range of interfaces and modalities prompts the search for seamless integration, aiming for an optimal interaction experience to enrich the coach–user relationship.

Several research projects integrate virtual coaching technology, such as the Wellbeing and Health Virtual Coach (WellCo) [9], which introduces an ICT-based platform centered on a virtual coach for well-being and health, emphasizing behavior change. This platform aims to facilitate crucial behavior changes for a healthier lifestyle through personalized intervention techniques, addressing three key conditions: capability, opportunity, and motivation. The platform ensures continuous monitoring of the user’s status and “Life Plan”, leveraging an affective-aware virtual coach and a team of multi-disciplinary professionals to enhance behavioral performance.

The HoloBalance project [10] introduces a novel and cost-effective virtual coach, incorporating a hologram-based surrogate balance physiotherapist, an augmented reality cognitive game, auditory exercises, and a physical activity planner. This comprehensive approach [11] provides personalized coaching specifically designed for individuals experiencing balance disorders associated with age-related sensory loss.

EMPATHIC [12] introduces a combination of multimodal face analytics, adaptive spoken dialogue systems, and natural language interfaces alongside non-intrusive technologies. This integration aims to extract physiological markers of emotional states in real time, providing support for both dependent aging individuals and their caregivers.

The Council of Coaches project [13,14] introduces CAs that enable multi-party interactions. The concept, known as the Council of Coaches, represents a groundbreaking virtual coaching model featuring multiple autonomous virtual coaches forming a personal council to comprehensively address the needs of older adults. This approach involves an open dialogue, allowing clients to collaboratively construct personalized plans for a healthier lifestyle with a selected group of coaches. The virtual coaches are embodied as conversational social characters.

The NESTORE Coach project [15,16,17,18,19,20] introduces a multi-domain personalized pathway for e-coaching, allowing users to integrate healthy activities. It utilizes automated monitoring and self-reporting through wearables, social beacons, cognitive games, and food photo analysis. 

CAPTAIN [21,22,23,24] utilizes augmented reality for non-invasive emotional, behavioral, and physiological data collection, offering motivational guidance for healthy habits. Supporting Active Aging through Multimodal Coaching provides a platform for well-being monitoring and assessment with minimal user input. The proposed technology aims to transform the living space of older adults into a seamlessly integrated assistant, leveraging projected augmented reality through micro-projectors to overlay contextualized information and instructions onto the actual environment, creating a transparent and user-friendly interface within the home.

The overarching goal of vCare (Virtual Coaching Activities for Rehabilitation in Elderly) [25] is to facilitate the recovery of active and independent life at home for patients undergoing rehabilitation from impairments or disabilities. This is achieved through the implementation of a smart virtual coaching system, ensuring rehabilitation guidance, and maintaining the continuity of care in the home environment and daily life. The project focuses on addressing two key challenges in the healthcare landscape: a participatory design approach driven by user needs and the personalization of care pathways facilitated by technology. Leveraging the rehabilitation setting as an opportune environment for prolonged user interaction enables both physicians and patients to contribute to shaping the system.

SAAM [26] is designed to assist the aging population residing at home, focusing innovatively and practically on ambient sensing and understanding user needs and preferences. It achieves effective coaching by utilizing the user’s social support networks [27].

The solutions presented illustrate promising approaches in the field of supporting older people for a fulfilling and independent lifestyle. Commonly used solutions include smartphone applications that offer virtual coaching for various aspects of daily living, along with audio/video systems that analyze user data to provide emotional support and wellness coaching. Emerging technologies such as micro-projectors and augmented reality are highlighted, creating tangible interfaces within home environments and serving not only healthy older users but also the rehabilitation at home of people with chronic diseases or physical ailments, such as problems with balance. Additionally, facial analysis, adaptive spoken dialogue systems, and natural language interfaces hold promise for engaging users and motivating them to achieve predefined goals. This reflects the evolving landscape of technologies dedicated to improving the well-being of the aging population through innovative and advanced approaches.

In Japan, a similar trend is observed in several projects focusing on exploring the use of new technology in a digitalized and connected society. The general goal is set in the context of the Society 5.0. framework, aiming to foster innovation and incorporate advanced technologies into society to support possibilities for anyone to enjoy a high quality of life. For instance, NEDO 2.0 “Future AI and Robot Technology Research and Development Project” (2015–2020) [28] focused on developing core technology and its “social implementation” to enable solving concrete problems by next-generation AI and robotics, while the METI/AMED Project “Promote the Development and Introduction of Robotic Devices for Nursing Care” (2013–2017) [29] aimed to develop prototype nursing care devices and determine guidelines and evaluation methods. A previous EU-Japan collaboration project, CARESSES (Culture-Aware Robots and Environmental Sensor Systems for Elderly Support) [30], focused especially on culturally capable robots that could interact appropriately in different cultural contexts and address user needs by managing sensor equipment, such as lighting and temperature, in the user’s connected environment.

As evident, ongoing and prospective research endeavors in virtual coaching systems are geared towards the integration of advanced patient monitoring, enhancing interactions between users and virtual systems, and, notably, enabling automated feedback and dynamically personalized interventions. These advancements are guided by personalized assessments derived from sensing data.

The e-VITA project stands out among the state-of-the-art initiatives by presenting a unique multiplicity across various dimensions, including various mobile and fixed sensors, robots, services, and interconnected processing steps. Notably, it integrates smart environmental sensing as a dialogue trigger and, in this way, creates dialogue scenarios adapted to the user’s context and status. This innovative approach introduces novelty by incorporating multimodal, multidomain, and multilingual elements, harnessing OpenAI’s ChatGPT within the virtual coaching framework [31]. Moreover, the project embraces a multicultural context, enhancing its distinctiveness within the field.

Table 1 provides a clear indication of which projects have contributed to specific aspects, facilitating a direct comparison of their contributions in various dimensions.

## 3. Architecture of the e-VITA Platform

The primary objective of the e-VITA platform is to furnish the necessary elements and functionalities for developing and running the e-VITA virtual coaching system, which aims to promote smart aging.

**Figure 1:**
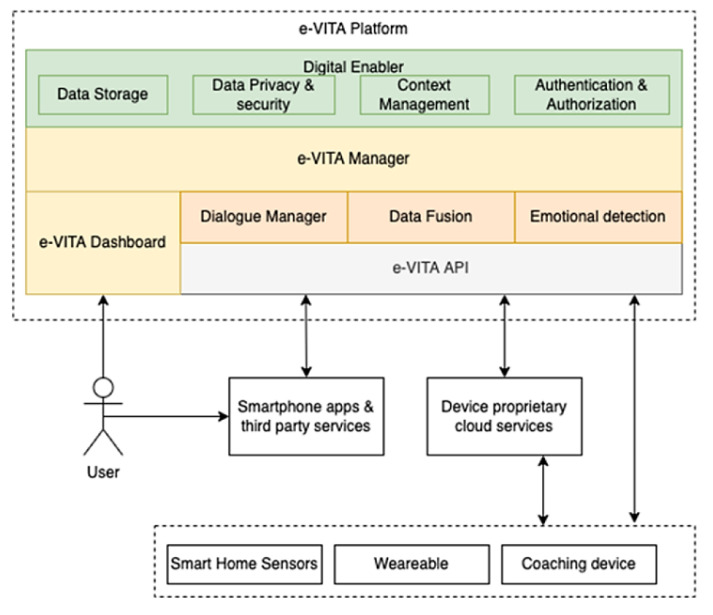
High-level overview of the e-VITA platform.

The design of the e-VITA platform considered various requirements and challenges specific to the project’s context. First, the e-VITA experimentation scenarios required the collection of data from heterogenous devices, including IoT sensors and smart devices from different vendors, each based on distinct data models and technologies. Additionally, the platform had to interact seamlessly with coaching devices such as robots and holograms, facilitating the transfer of user messages to the dialog system and receiving corresponding answers. Lastly, the e-VITA platform needed to serve as the backbone for the entire coaching system, providing general capabilities for storage, security, and overall communication with external systems, with a particular focus on addressing privacy aspects related to data protection and consent management. In response to these requirements, a modular and extensible architecture was devised, as illustrated in Figure 1.

The e-VITA platform incorporates a set of software assets, either provided by e-VITA consortium partners or sourced from existing open-source projects and initiatives. One of the objectives was to maximize the reuse of existing software products, reduce the effort required for new implementations, and focus on the integration process. This approach facilitated the rapid implementation of the coaching system by leveraging mature software assets and capitalizing on the partners’ expertise in their usage and customization. The architecture components exhibit different levels of maturity, with some evolving during the project within the technical work packages. The e-VITA platform connects and integrates the various device types within the system, including smart home sensors (such as temperature and humidity sensors and intrusion sensors), wearable devices (including smartwatches, smart bands, and smart rings), and coaching devices (such as robots). Detailed information regarding the specific devices integrated into the platform can be found in Section 4. Communication between devices is established through e-VITA APIs, a set of RESTful HTTP-based APIs that ensure data transmission with essential security procedures, such as authentication. The API-based approach streamlines interaction with heterogeneous devices and allows for easy extensibility of the system for future devices. Additionally, these APIs serve as the primary interface for accessing both current and historical data, as well as platform features, from mobile applications or third-party services.

Playing a central role, the e-VITA Manager orchestrates the integration between devices and specific e-VITA modules, including the Dialogue Manager, Data Fusion, and Emotional Detection. It manages connections with the Digital Enabler (DE) [6], a platform based on FIWARE technology, offering functionalities for security and data management. The DE provides features such as user and service authentication and authorization, context management through the FIWARE Context Broker, historical data storage from devices, and data privacy and security measures. Serving as the primary user interface for accessing platform features, the e-VITA Dashboard completes the key components of the platform. 

**Figure 2:**
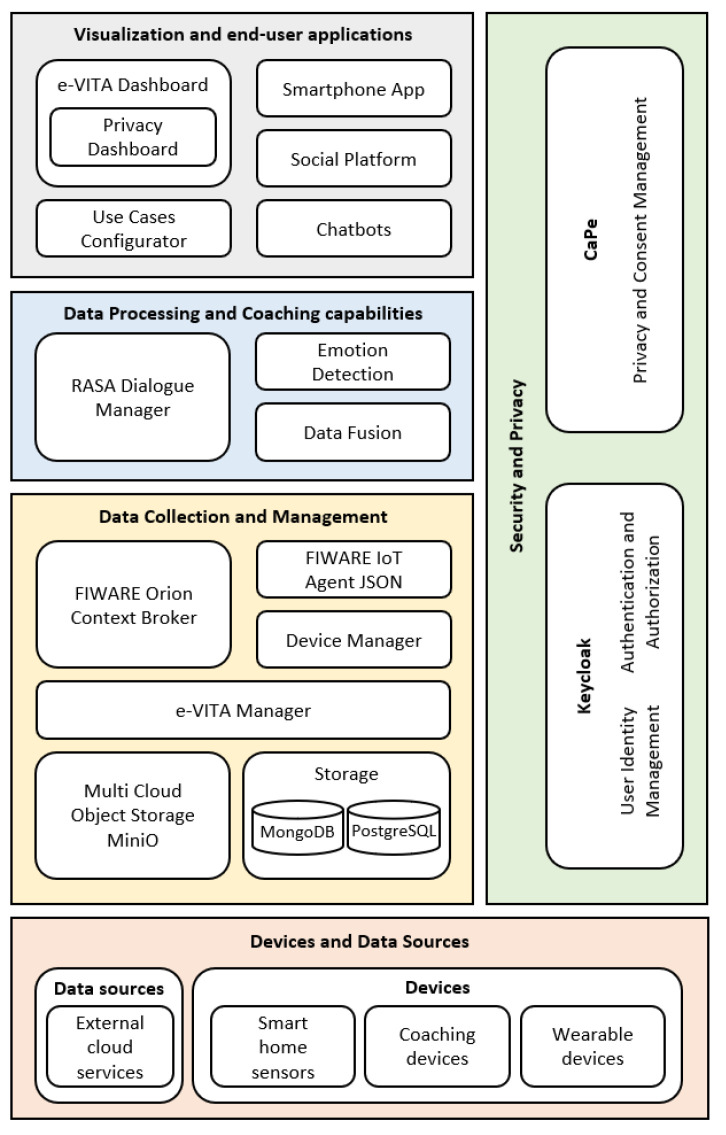
e-VITA platform architecture.

Figure 2 offers a detailed view of the e-VITA architecture based on functional levels:

1. Data Collection and Management: This layer encompasses modules for managing context data, processing, and storage, facilitating real-time orchestration between applications, ensuring data interoperability, and harmonizing information from devices and external systems. The e-VITA platform supports different types of data storage, including Object Storage, SQL/NoSQL databases, and storage for linked data like triple stores;
2. Data Processing and Coaching Capabilities: this layer manages an interactive speech-based Dialogue Manager for active user interaction and handles data processing to extract valuable insights;
3. Visualization and End-User Applications: this layer generates visual dashboards based on collected data from other components, enabling visualization capabilities for the e-VITA platform’s overall data and allowing end-user applications to utilize platform services (e.g., configuration of device connections, interaction with the dialog manager via web interface, etc.);
4. Security and Privacy: this layer focuses on security and privacy features, including identity management, authentication, authorization, pseudo-anonymization, and consent management, controlling access to data and services;
5. Devices and Data Sources: this layer encompasses devices and data sources interacting with the main e-VITA platform, serving as interfaces for end users accessible through different modes of interaction, and systems providing data to the platform.

## 4. Platform Components

The e-VITA platform incorporates various modules, and this section delineates the key ones essential for understanding the functionality of the platform.

### 4.1. Devices and Data Sources

The e-VITA platform integrates diverse sensors and coaching devices to capture and assess different aspects of user behavior, emotions, and physiological indicators. These sensing technologies fall into three primary categories:

- User-related devices: These devices, intended for users to wear, primarily focus on sensing physiological parameters. They can monitor vital signs and other relevant health-related data. Examples include wearable fitness trackers, heart rate monitors, and sleep-tracking devices;
- Environmental devices: Positioned within the household, these devices measure physical variables contributing to comfort levels and Indoor Environmental Quality (IEQ). They monitor factors such as temperature, humidity, air quality, and lighting conditions, providing insights into the overall environmental circumstances affecting user well-being [32];
- Home-based devices: Installed within the user’s home, these devices monitor the user’s conduct and activities, offering insights into daily routines, movement patterns, and interactions with their surroundings. Examples include motion sensors and intelligent appliances that aid in recognizing activities [33,34].

Table 2 lists the aforementioned devices, outlining their main functionalities. The selection of specific devices was influenced by varying market availability, with some devices chosen for European use and others selected to measure the same quantities in Japan.

**Figure 3:**
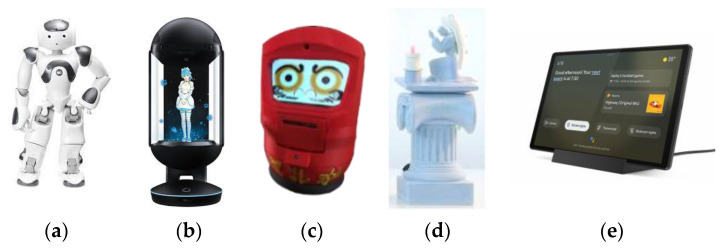
e-VITA platform coaching devices: (**a**) NAO robot; (**b**) Gatebox hologram; (**c**) DarumaTO; (**d**) CelesTE; and (**e**) Tablet.

The coaching devices integrated into the e-VITA platform are presented in Figure 3 and include the following:

1. NAO, a compact humanoid robot, is widely employed in studies centered on human–robot interaction [35,36,37]. The NAO robot serves as a versatile platform for diverse interactive tasks, using speech, gestures, and other interactive behaviors to engage users. Its role within the project involves acting as a coach, providing guidance and support to elderly users;
2. Gatebox, a device that generates a visual representation (hologram) of a virtual coach through a 3D effect [38]. The Gatebox hologram projects an avatar’s image, enabling an interactive coaching experience. The virtual coach communicates with users, delivering guidance and relevant information;
3. DarumaTO [39,40,41], a social robot fashioned after a traditional Daruma doll from Buddhist and Shinto traditions. The DarumaTO robot resembles the doll’s appearance and interacts with users via facial expressions and speech. It serves as a companion and coach, offering emotional support and assistance to older users;
4. CelesTE, an interactive small angel statue designed specifically as a spiritual companion for Christian Catholic seniors, inspired by the existing SanTO robot [42,43,44]. CelesTE engages with users, providing companionship, guidance, and spiritual support;
5. Tablet with a built-in Google assistant that serves as a coaching tool, delivering personalized information and reminders as well as engaging in voice-based interactions with older users [45].

Some of the coaching devices were selected from the market, including NAO and the tablet. NAO was preferred over other humanoid robots, such as Pepper, because it was more compact and easily accessible for the project’s interaction scenarios (home setting), offering advantages in terms of accessibility and user engagement. In fact, in several tests conducted over the years, NAO has been judged as one of the best companion robots, cute, friendly, and intelligent [46]. In addition, some devices were developed by project partners, specifically CelesTE and DarumaTO. Some devices, on the other hand, were chosen as commercial products of project partners; this is the case of the Gatebox device, introduced by the respective partner and adapted for use in the e-VITA project.

### 4.2. Data Collection and Management

#### 4.2.1. Device Manager

The Device Manager (DEMA) component, part of the Digital Enabler (DE) ecosystem platform, facilitates the connection of sensors and IoT devices to the platform via FIWARE’s IoT Agents [47]. It is a Java-based component designed for easy management of sensors and IoT devices, enabling registration, management, and monitoring. The DEMA offers REST APIs for publishing, deleting, and obtaining device information, providing comprehensive and centralized management. While the DEMA backend REST APIs are used in the e-VITA platform, final users manage devices directly from the User Interface (UI) in the e-VITA Dashboard. The IoT DEMA supports different transport protocols and data formats, relying on FIWARE IoT Agents, including the IoT Agent JSON. It can be easily extended with a new ad hoc IoT Agent for unsupported protocols and data formats.

#### 4.2.2. FIWARE Orion Context Broker

The FIWARE Orion Context Broker [48] serves as an NGSI server implementation within the e-VITA architecture. It manages context information collected from devices or sensors, handling updates, queries, registrations, and subscriptions. In the e-VITA platform, the Context Broker harmonizes raw data coming from devices via publish–subscribe APIs. It is primarily involved in receiving and storing the latest measurements from sensor devices, as well as reading the latest measurements of a specific device. The Context Broker plays a vital role in providing context information for external client applications and the e-VITA Dashboard.

#### 4.2.3. FIWARE IoT Agent JSON

The IoT Agent JSON [16] facilitates the transmission of data from a group of devices to a Context Broker, managing it through their native protocols. In the e-VITA context, the IoT Agent JSON acts as a bridge between JSON-based protocols and the NGSI interface of a Context Broker (i.e., FIWARE Orion Context Broker). It allows device measurements to be stored within the Context Broker, supporting the ETSI NGSI-LD API protocol based on simple single-level JSON object encoding.

#### 4.2.4. Multi-Cloud Object Storage MinIO

For Object Storage, the e-VITA platform utilizes MinIO Object Storage, compatible with the Amazon S3 cloud storage service API [49]. This open-source distributed Object Storage server supports different typologies of data storage, ensuring high performance. Each registered user on the e-VITA platform is associated with a Bucket in MinIO, organizing objects containing historical data (all measurements sent by devices to the platform) from registered devices.

#### 4.2.5. Data Storage

Data storage involves storing information (data) on the e-VITA platform. The e-VITA Manager manages the proper storage and handling of all necessary information for the platform (information on users registered on the platform, measurements sent by devices, messages exchanged between the user and the Dialogue Manager), utilizing both relational and non-relational databases. The open-source MongoDB database is adopted [50] for its flexible schema, which stores records as BSON-described documents. PostgreSQL [51], a strong object-relational and open-source database system, is also used for storage. PostgreSQL provides transactional support and features like atomicity, consistency, isolation, and durability. Additionally, it offers features like automatically updated views, materialized views, triggers, foreign keys, and stored procedures. This database system is engineered to manage a diverse array of workloads, whether they involve a single machine, data warehouses, or web services with numerous simultaneous users.

#### 4.2.6. The e-VITA Manager

The e-VITA Manager functions as the central and key component of the e-VITA platform, developed within the project to orchestrate integration among devices and e-VITA-specific modules. It plays a crucial role in managing the heterogeneity and complexity of e-VITA’s technological assets, dealing with multiple devices and software components across Europe and Japan, each based on different technologies and standards, which made the design of a coherent technical architecture a challenging objective. It manages the different components and their relationships in terms of functionalities and data exchange processes. Therefore, the e-VITA Manager is the backbone of the e-VITA coaching system, providing all the necessary capabilities to enable communication and integration among different smart devices (e.g., sensors and robots) to harmonize and manage the collected data and to provide different data processing and analytics capabilities needed for the coaching functionalities. The e-VITA Manager is the back-end component that is developed via a microservice architecture. It exposes its functionality through a set of REST APIs [52] that allow devices to interact with the system and allow external services to use the basic functionalities and data collected by e-VITA in compliance with security and privacy requirements. Specifically, through a set of appropriately implemented connectors, it allows the correct integration of IoT gateways, wearables, devices, and robot sensor data. The set of REST APIs exposed by the component can be consumed by any client. It works as a middleware component to interact with the Device Manager of the Digital Enabler. Some devices can directly send data to the e-VITA platform, while others are configured to send and store the detected measurements in the vendor’s cloud service. Devices can be grouped into two categories. From the point of view of the interaction with the e-VITA platform, the differences between the two categories are related to how the e-VITA platform accesses the device data; thus, a specific solution is provided for each category. The two categories are the following:

- PUSH: the device itself sends data to the platform directly or via a dedicated gateway. The e-VITA system facilitates data transmission for PUSH devices, catering to two specific types: smart home sensors that transmit measurements and coaching devices (robots) that deliver audio messages to the e-VITA Manager. In the first case, the device initiates the process by invoking the specific REST API exposed by the e-VITA Manager; the latter verifies the device’s registration on the platform and then forwards the measurements to the IoT Agent JSON. This agent is responsible for storing the data in the Orion Context Broker and archiving it within MinIO Object Storage. In the second case, a coaching device sends audio messages to the e-VITA platform to be processed by the RASA Dialogue Manager. The user interacts with the coaching device using voice commands, and the device converts the speech to text, employing specific speech-to-text functionality or services. After processing, the textual response from e-VITA is transformed back into audio for playback by the end user;
- PULL: measurements acquired by the device are stored in the vendor’s external cloud and made accessible via a proprietary, secure REST API. The e-VITA platform periodically retrieves data from PULL devices via API calls. The measurements from PULL devices are managed and stored in the vendor’s clouds. A scheduled thread within the e-VITA Manager is registered for each PULL device, ensuring timely data retrieval. The vendor’s token is obtained for validation and is then utilized to fetch measurements from the external cloud. After successful validation, the external cloud provider returns the measurements to the e-VITA Manager. Subsequently, the Manager propagates the data to the Digital Enabler within the Orion Context Broker through the IoT Agent JSON and, finally, stores the information in MinIO Object Storage.

**Figure 4:**
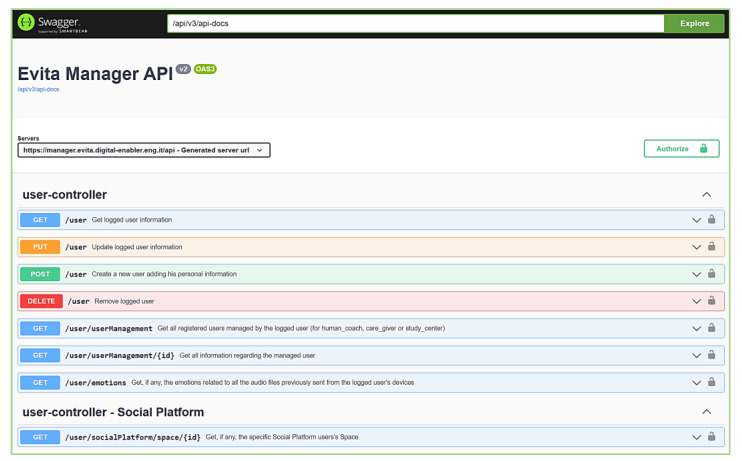
e-VITA Manager—Swagger.

In addition to this main feature for managing device measurements, the e-VITA Manager exposes many other back-end operations, implemented via REST APIs, for registering and managing users and devices and for communicating with other components. The e-VITA Manager provides the Swagger (Figure 4) that documents these functionalities.

Swagger is an available and attainable online resource that adheres to the OpenAPI specification [53]. The Swagger lists all available APIs with their detailed description, all supported operations, the input parameters of each API, and the returned value; it notifies whether the API needs authorization and even the terms, contact information, and license for the use of the APIs. Through Swagger, it is also possible to use the API directly by freely entering input parameters.

Detailed additional information on e-VITA back-end functionalities can be found in the project deliverable “D7.4 e-VITA Platform Architecture—Final Version” [4].

### 4.3. Data Processing and Coaching Capabilities

#### 4.3.1. Rasa Dialogue Manager

The interaction with the user is managed by the Dialogue Manager component, which is built based on Rasa Open-source Conversational AI version 3 [54,55]. The dialogue system also uses LangChain v0.1.1 [56], OpenAI ChatGPT model gpt-3.5-turbo [57], PyPDF v3.17.4, and FAISS v1.5.3 [58]. 

**Figure 5:**
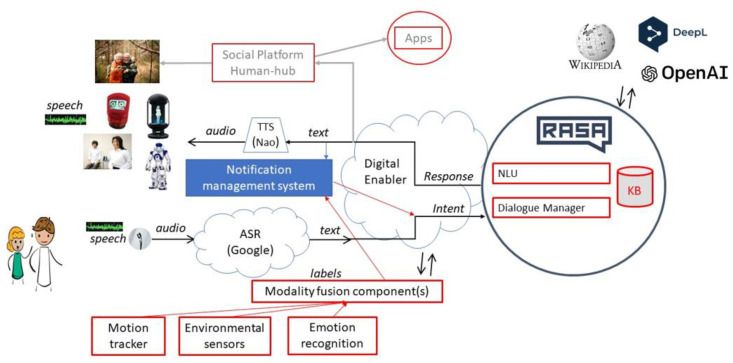
Overall system architecture for dialogue model integration.

Figure 5 shows the overall architecture and integration of the dialogue component into the overall system. The core Dialogue Manager adapts to the latest technologies, including large language models, the use of APIs and devices, NLU components, and response selectors. It customizes Rasa’s base pipeline to take care of user input processing, dialogue states, and response generation.

Our Dialogue Manager consists of 304 intents, which are categorized across multiple domains, i.e., exercise, nutrition, daily habits, news, etc. The main idea of including these intents is to identify user utterances and respond accordingly.

The API hub includes information from the sensors, the emotional recognition system, motion trackers, and environmental sensors. The user’s personalized information drives the dialogue through specific, individualized routes, with such information also contained in the API hub. A notification management system has also been added to the whole architecture, which allows external conditions to start dialogues not initiated by the user. These conditions are based on sensor status or timing, i.e., the system can prompt the user with outside exercises every morning at 8 am or remind them to drink enough water if the weather outside is too hot.

**Figure 6:**
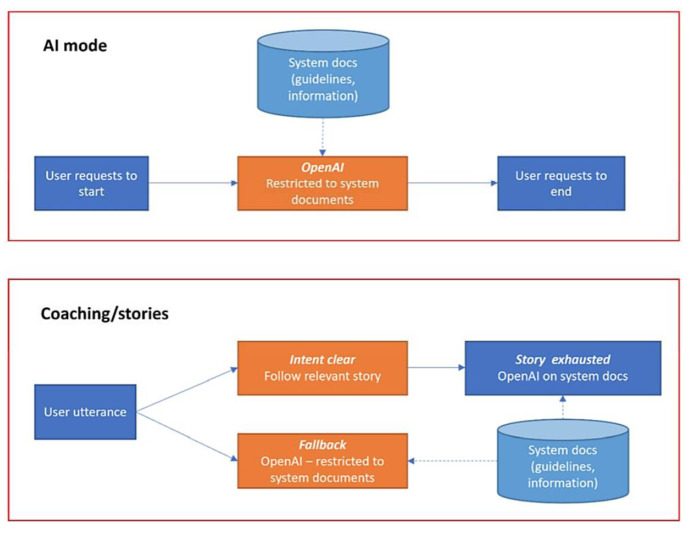
OpenAI access in the EU. The AI mode (at the top) is requested and ended by the user. In normal “coaching” mode (bottom of the figure), the OpenAI API is only invoked when the user intent is unclear or a scripted story has been exhausted. In both cases, the call always uses the LangChain framework to restrict the conversation to the controlled domain.

**Figure 7:**
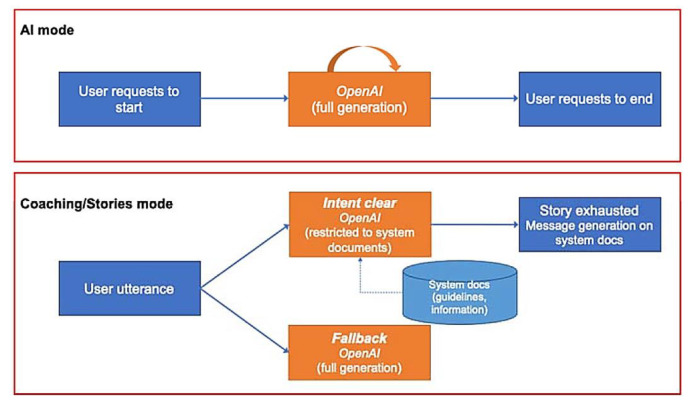
The OpenAI access in Japan. The system leverages the OpenAI GPT-3.5 capability to generate responses both in the AI mode and coaching fallback modes. The less restrictive policy is allowed by the ethical approval of the use of generative AI on the Japanese side.

The European and Japanese systems have slightly different architectures regarding the use of OpenAI language models. When producing responses driven by the OpenAI API, the European system generates such responses using the Retrieval Augmented Generation (RAG) approach with LangChain (see Figure 6). The reason is that the EU system cannot use “unrestricted” access to the OpenAI API due to ethical considerations, and RAG is a common way to provide truthful responses and bypass the hallucination tendency of the direct use of the OpenAI API. This approach helps reduce ChatGPT hallucinations by relying on a curated set of domain-specific PDF documents. It is complemented by the use of carefully crafted instructions (prompts) provided to the model, which direct the generated content to be restricted to the information supplied in the documents. This focused dataset provides contextual guidance as part of the input prompt, ensuring responses align closely with accurate information from the documents. The approach aims to maintain truthfulness and accuracy, address ethical concerns, and minimize the risk of generating misleading content in responses. On the Japanese side, however, OpenAI’s ChatGPT can be used directly to handle domain-specific queries (see Figure 7). 

The OpenAI ChatGPT model *gpt-3.5-turbo* is utilized via the API to support a fallback functionality of Rasa, exploring the generative power of ChatGPT in cases where an appropriate user intent is not found, as well as in a separate AI mode that the user must activate and deactivate. (1) Fallback mode: when the user intent is not properly identified, i.e., the predicted intent has a confidence lower than a set threshold, the OpenAI model takes over and generates the answer from the provided documents. In our case, the threshold value is 70%; (2) AI mode: the user can activate this mode by a predefined utterance, i.e., “Activate Artificial Intelligence”.

On the European side, the fallback and AI mode are both based on the LangChain functionality, due to ethical reasons. An important aspect of the Japanese system is to make sure that the users are aware that they are talking to ChatGPT with less reliable answers than if the system implemented a safer LangChain approach. However, further studies and comparisons between the two approaches are being discussed in the project. 

To analyze the usefulness of each fallback, the system generates logs of the interactions, including the text of the responses, intents, and fallback types.

**Figure 8:**
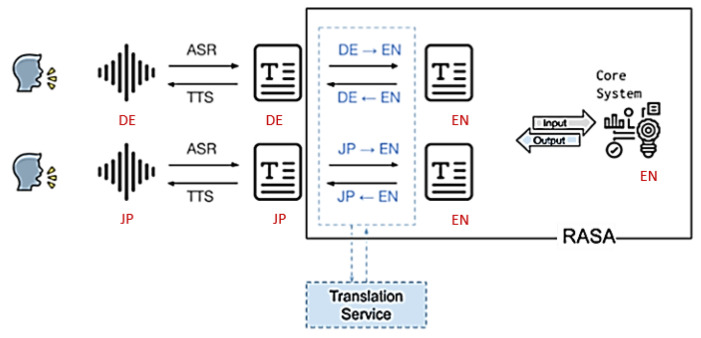
Multilingual translation API.

The system has been developed to cater to different languages spoken in different partner countries (German, French, Italian, Japanese, and English for development). The core system itself runs in English but relies on the external translation service DeepL API [59]. This is used to translate the user’s speech transcript into English and, again, the system’s English output back into the user’s language. The output is then spoken using the language-specific text-to-speech (TTS) component. Some manual revision and tweaking have been performed to guarantee the appropriate style and politeness level, especially for the Japanese language. Figure 8 exemplifies the use of translation in the system.

The e-VITA coach has an ambitious list of desired interaction functionalities. It is intended to provide truthful information to the user on various topics relevant to the older user’s daily life activities, e.g., cognitive and physical training, spiritual guidance, and friendly chatting. Proactive recommendations depend on the user’s emotional and physiological state, history of daily activities and movements, and environmental sensor information. Ultimately, the coach is expected to show capability for advanced coaching interaction, following a motivational coaching cycle that takes into account the user’s motivation and readiness level.

The evaluation of a preliminary system focused on user expectations and showed that users have a positive opinion of the coaching system and strongly desire the information to be tailored to their daily lives [35]. More aspects of the system are detailed in [5,60,61], underscoring the testing methodology that involves a thorough process of gathering requirements for the virtual coach. This process incorporates interviews and participatory design in real-life settings, reflecting a user-centric approach that ensures alignment with the specific needs and preferences of older adults.

Building upon these foundational aspects, the current work introduces significant advancements in the new platform, characterized by more complex knowledge modeling and the creation and seamless integration of knowledge bases that contain the pertinent knowledge of the domain. This integration extends beyond the dialogue system, encompassing additional components such as new knowledge bases, sensor data, and insights from an Emotion Detection System. These enhancements demonstrate a progression towards a more sophisticated and integrated system, showcasing the evolving nature of the virtual coach platform.

#### 4.3.2. Emotion Detection System

Affective computing is crucial to the development of empathic technologies capable of understanding and analyzing affective and expressive behavior [62], which is a fundamental aspect of the e-VITA platform. The platform employs the Emotion Detection System (EDS), which is dedicated to identifying prevalent basic emotions such as anger, disgust, fear, happiness, neutral, sadness, and surprise, utilizing predominantly spectral energy domain features in speech [63]. This specialization enables the detection of nuances and classification of emotions during voice interactions between the coaching system and older adults, aiming to optimize user interactions [64]. The EDS features a multilanguage Speech Emotion Recognition (SER) model, which is trained to label audio signals with one of the seven basic emotional classes. The model leverages a comprehensive database of 25,596 acted/non-acted emotional speech samples across English, Italian, Japanese, and German languages (Table 3). The distribution of emotion categories in the overall dataset is shown in Table 4. 

SER, a deep learning model, specializes in analyzing audio signals to predict emotion classes. It discerns intricate patterns in the pre-processed data using feature vectors (such as log-Mel spectrograms), ensuring high predictive accuracy. These predictions are integrated into the dialogue system, enabling personalized interventions and feedback based on the detected emotions. This enhances the responsiveness and effectiveness of user interactions [62]. The integration of SER demonstrates the ability of EDS to not only understand but also appropriately respond to emotions, addressing the inherent complexities and variability in speech emotion recognition tasks. 

**Figure 9:**
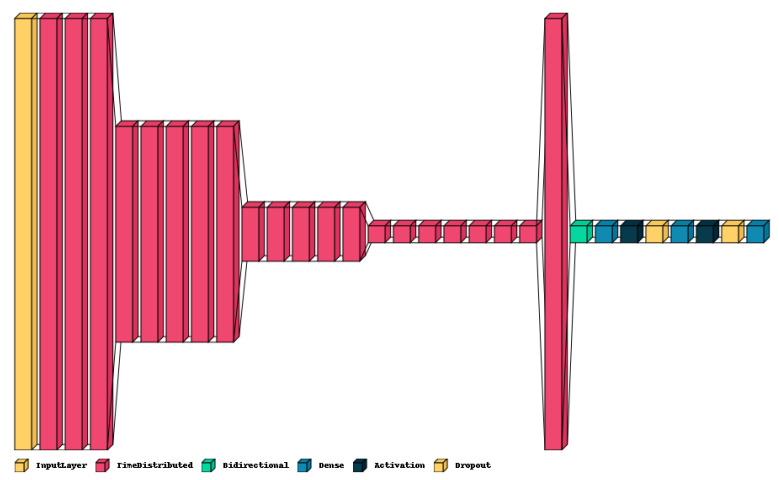
The SER model architecture, featuring an input layer, four LFBLs with CNN, normalization, activation, pooling, and dropout, a flattening fully connected layer, an LSTM layer, two dense blocks, and an emotion class output layer.

The model architecture, illustrated in Figure 9, is described as follows:

- It processes the audio data through four sets of specialized blocks, termed Local Feature Learning Blocks (LFLBs). Each LFLB consists of the following layers:
  ○ A two-dimensional Convolutional Neural Network (2D-CNN) extracts temporal and spectral features from the input spectrogram;
  ○ A batch normalization layer normalizes the output of the CNN layer;
  ○ An activation layer;
  ○ A max pooling layer down samples the 2D-CNN output, reducing parameters and improving computational efficiency;
  ○ A dropout layer is incorporated to minimize overfitting.
- The output from these layers is subsequently transformed into a simplified format using a flattened layer for easier processing by subsequent layers;
- A specialized layer, known as bidirectional Long Short-Term Memory (LSTM), is used to analyze the data sequence and identify evolving patterns over time in the audio signal. This layer takes a sequence of spectrogram frames as input and learns to model sequential dependencies among these frames, capturing temporal features;
- Additional dense layers are incorporated to further refine and generalize the understanding of patterns and relationships within the data;
- Finally, the model uses an output layer to calculate the probabilities of the audio belonging to each of the seven emotion classes.

The performance of the SER model is assessed using precision, recall, and F1-score as metrics. Detailed performance metrics for each emotion category are provided in Table 5. 

**Figure 10:**
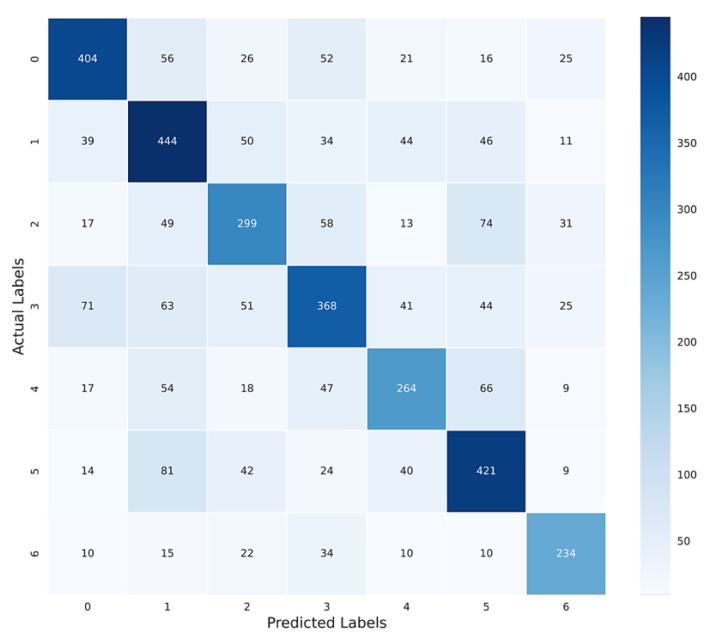
Confusion matrix of SER: the labels correspond to emotion class (0: anger, 1: happiness, 2: sadness, 3: neutral, 4: fearful, 5: surprised, and 6: disgust).

The classification report indicates a relatively strong performance of the model for certain emotions, notably anger and surprise, while demonstrating a relatively lower performance for fear and neutral emotions. The average accuracy of SER is 62%, which represents the average of the diagonal of the confusion matrix (Figure 9). It is similar to the overall accuracy, but it considers the balance between classes. While accuracy gives an overall estimation of the model’s performance in all classes, the F1-score gives an estimation of the model’s performance in each individual class in terms of precision and recall. Thus, due to the imbalanced dataset, the F1-score can provide a more realistic evaluation of the model’s performance in each class. Additionally, the presented confusion matrix (Figure 10) provides a visual representation of both correct and incorrect predictions made by the model across each emotion category. It helps to identify the specific emotion categories where the model has difficulty distinguishing and which categories are more likely to be misclassified. It is essential to interpret the performance evaluation with caution, considering the dataset’s characteristics and the inherent complexity of speech emotion recognition tasks, influenced by factors like language, data quality, and speaker variability, which can impact the SER model’s performance. 

The e-VITA project’s EDS relies on prosodic features from end users’ recorded speech, with the algorithm learning from extensive training databases containing various emotional speech samples. Combining existing corpora is challenging due to the lack of standardized assembly methods, requiring meticulous assessment and standardization efforts.

Recognizing cultural variations in speech-based emotional expressions, the search for EDS training data includes speech data from all target languages (German, Japanese, French, and Italian) within the consortium. However, limited data from older adults (the project’s target population) may impact algorithm generalizability. Despite minimal research on emotional prosody differences between younger and older adults, efforts are made to include data from older adults, and age metrics for all corpora are reported to mitigate age-related cross-corpus generalizability risks.

In the search for emotional speech corpora to train the EDS, various parameters were considered. Data were sourced through direct contact with known providers, literature research, and existing assets. To be eligible for inclusion, a dataset had to contain samples in one of the target languages (German, Japanese, French, or Italian) or English, which is the consortium’s lingua franca. The corpus encompassed recordings of acted, naturally expressed, and induced emotions.

Key parameters identified for each corpus included publication year, spoken language, age demographics (range, mean, and standard deviation), included emotions (with a focus on the most prevalent ones), the number of samples per emotion, recording of video data in addition to audio, total corpus size (files, utterances, speakers, and duration), audio sampling rate, mean speaking duration per file, mean data units per participant, emotion origin, recording under EU H2020 or Japanese projects, licensing details, acquisition methods (e.g., download availability), and scientific reporting if applicable. This detailed approach aimed to ensure a thorough understanding of each corpus’s characteristics for effective use in training the EDS.

In ensuring the quality of emotional speech corpora for training the EDS, a standardized approach was employed across all source corpora. Quality checks were performed through a representative subsample of recordings, evaluating whether each dataset was suitable for emotional computing. The subsamples included the ten smallest and ten largest recordings (by file size), along with an additional pseudo-randomized subsample of up to 50 files, depending on the total file count. Subsamples were constructed to ensure the inclusion of each speaker, each recorded emotion type, and each experimental task type. Each of the 70 selected files underwent a thorough assessment, considering parameters such as the presence of sound, the existence of background noises, the number of recorded speakers, the match between the documented gender (in metadata) and the recorded voice, alignment with the documented task category, and consistency with the documented specific task. 

In terms of database properties, a total of 59 corpora have been identified for the EDS (15 in English, 18 in German, 11 in French, 11 in Japanese, and 4 in Italian). Out of these, 11 corpora cannot be obtained, and 28 are pending replies from suppliers. Challenges in obtaining corpora include high licensing costs, authors’ unwillingness to share due to corporate interests, and complex legislative regulations. Regarding the size of corpora, not all are equally valuable, with only a few containing a substantial number of samples. On average, corpora include 1684.93 files and are comprised of 47.38 speakers. The average file count per language is 2119.75 for English, 1416.33 for German, 301 for French, 1060.75 for Japanese, and 2791.5 for Italian. The corresponding speaker averages per language are 51.23 for English, 32 for German, 99.6 for French, 14.5 for Japanese, and 48.33 for Italian.

**Figure 11:**
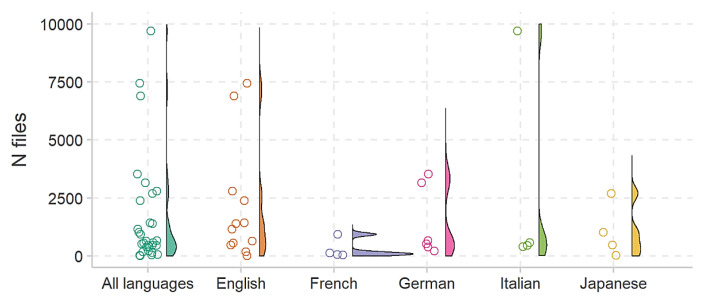
Number of files per corpus, with circles representing individual corpora and curves visualizing the distribution (density) across corpora. Non-obtainable corpora have been excluded from the figure.

**Figure 12:**
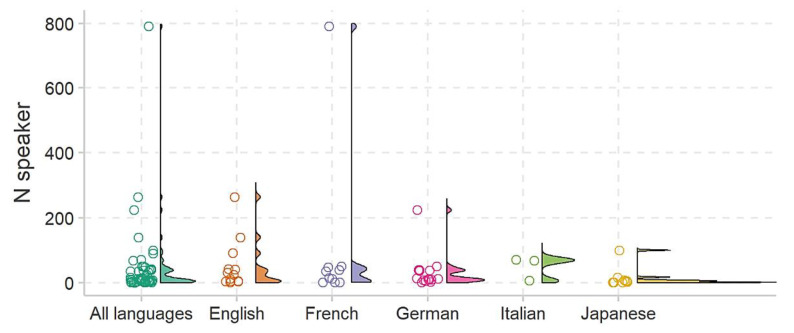
Number of speakers per corpus, with circles representing individual corpora and curves visualizing the distribution (density) across corpora. Non-obtainable corpora have been excluded from the figure.

The distributions of file counts and speaker numbers are illustrated in Figure 11 and Figure 12, respectively. It is essential to note that these corpora details are crucial considerations in the development of the EDS in the e-VITA project.

On average, the identified corpora for the EDS have a total recording duration of 3.58 h (SD = 4.41). The average durations for English, German, French, Japanese, and Italian corpora are 4.83 h, 3.68 h, 1.51 h, 4.95 h, and 2.32 h, respectively, excluding corpora that are not obtainable.

The majority of emotional speech corpora were obtained from younger adults, as reflected in a grand average mean age of approximately 30.17 across all corpora. Individual ages within the datasets ranged from 10 to 76 years, indicating some inclusion of older adults, although the predominant source of data comes from younger individuals. This age distribution suggests a potential imbalance in age representation within the corpora, with a focus on the younger adult demographic.

**Figure 13:**
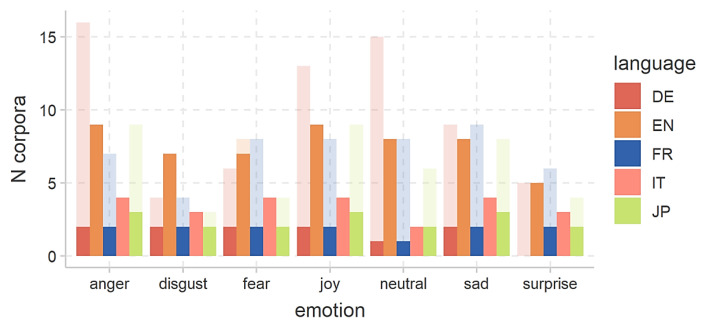
The number of corpora per emotion and language, with transparent bars showing the data of all requested corpora and solid bars representing already obtained data. Non-obtainable corpora have been excluded from the figure.

Corpora of emotional speech in the EDS are obtained through diverse methods, reflecting a lack of standardized recording procedures. This results in significant variations in the specific set of emotions included in each corpus. Figure 13 visually depicts the number of corpora by language and emotion, with slightly transparent bars representing the total number of corpora in the database and solid bars indicating those already obtained. Notably, data for all targeted emotions (anger, disgust, fear, joy, neutral, sad, and surprise) across all languages is available. The figure highlights a larger amount of English data, potentially attributed to extensive research in English-speaking countries.

While appropriate training corpora for all target languages have been identified and obtained, pending permissions, especially for Japanese corpora, pose a concern, impacting the potential performance of the e-VITA coach’s EDS. Despite this, the consortium believes that sufficient training data have been acquired, and additional data acquisition plans are outlined for potential insufficiencies or generalization issues, underscoring the ongoing nature of the project.

#### 4.3.3. Data Fusion Platform

The main objective of the Data Fusion Platform (DFP) is to enhance the quality of information by focusing on factors like accuracy and specificity and utilizing various multimodal data sources. In our specific context, the objective is to provide human activity recognition (HAR). Our data sources encompass inertial (accelerometer, gyroscope, and magnetometer) and location (latitude, longitude, altitude, and speed) data from smartphones, motion or intrusion detection data from Delta Dore and EnOcean sensors, and indoor climate data from Netatmo and EnOcean devices. 

The DFP is a web-based, FIWARE-standard platform designed to collect and process several data sources. It must be capable of batch or real-time processing. In this paper, we define real-time processing as “Process data faster than a significant change in input data”. However, this system is not a single-hosted system; it must be transferable and customizable to fit all data ingestion issues. Thus, the DFP is deployed under Docker to ensure operating system compatibility and maintainability.

Moreover, the DFP platform possesses two levels of security. The first level is architectural protection, which prevents unwanted users from reaching the system. This part runs under the OAuth2 protocol. The second level is data protection in case of failure of the first level. This is RSA-based data encryption for confidential information stored in databases. 

The components of the DFP can be sorted into the following categories:

- Core: data centralization and knowledge propagation;
- Dispatcher: open port reduction—it redirects to a specific service according to the user request;
- Central security: manage security at a global stage (all the platforms);
- Agent security: manage security at a local stage (dedicated to a specific component);
- Backend: customizable component;
  ○ Collector: collect data from sources.
  ○ Compute: process data (GPU capabilities).
  ○ Connector: login provider.
- Persistent: data persistence;
  ○ Database: store data on SQL or NoSQL logic;
  ○ Object Storage: store data as an object.

Previously, we stated that the platform follows the FIWARE standard. This comes from the core, central security, and agent security components. In fact, it uses FIWARE Orion Context Broker, FIWARE Cygnus for data historization, FIWARE STH-Comet for time series query, FIWARE Keyrock, and FIWARE pep-Proxy. This technology has a double advantage: on the one hand, it directly handles interoperability between data sources; on the other hand, it guarantees compatibility with the Digital Enabler.

Backends can be deployed as data collectors, data processors, or even login portals. The only specification is about communication and is referred to as Web Server Gateway Interface (WSGI) or Asynchronous Server Gateway Interface (ASGI). Obviously, according to this specification, the backends are developed with Python. Thus, with WSGI, we can get a REST API, and with ASGI, we can get WebSocket communication. Another aspect is that the backend can easily access the GPU from the host. 

**Figure 14:**
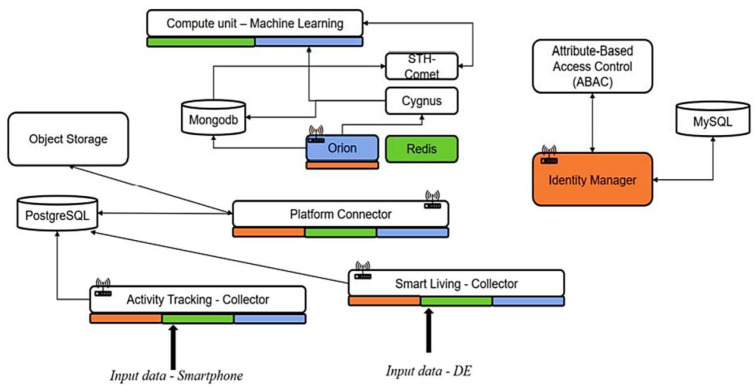
Basic DFP architecture.

To visualize the architecture, we propose the simplified schema in Figure 14. A color code is introduced to facilitate readability. Each service—Orion, Redis, or Identity Manager—with a color indication at the bottom is linked to the service with the same color.

To introduce a smart interaction between the Dialogue Manager (DM) and the output of the DFP, which would consist of triggering relevant dialogues according to the user’s contextual situation, we have retained and used Perseo as an Esper-based Complex Event Processing (CEP) software, developed by FIWARE and also provided by the Digital Enabler. Esper offers a language called Event Processing Language (EPL) that implements, by extending it, the SQL standard and enables rich expressions about events and time.

Perseo is intentionally designed to maintain full compliance with NGSI-v2 standards. It employs NGSI-v2 as its communication protocol for handling events, ensuring seamless and effective collaboration with Context Brokers like Orion.

Perseo operates on a simple concept: it listens for events originating from contextual information, searching for patterns described by predefined rules. These rules enable Perseo to respond promptly by triggering actions.

By making use of the notification mechanism, clients can instruct the Orion Context Broker to notify Perseo about any changes in the entities they are interested in (Event API). Subsequently, rules within the CORE Rule Engine can be easily managed using any REST client capable of programmatically accessing Perseo’s Rule API. These rules are designed to identify patterns that, when detected, trigger actions that initiate communication with RASA to initiate dialogues.

### 4.4. Security and Privacy

The e-VITA architecture provides different functionalities to ensure security and privacy, adhering to legal frameworks such as GDPR and APPI. User data management within the e-VITA platform covers different aspects, including the creation of user accounts, their association with deployed devices, and subsequent data analysis. The security and privacy layer incorporates features like identity management, authentication, authorization, pseudo-anonymization, and consent management for data and services. Keycloak serves as the identity provider, functioning as an Identity and Access Management tool for user authentication and management. Keycloak offers flexible customization of user interfaces for login, registration, administration, and account management. When users access the platform through the UI, they are redirected to Keycloak’s login form for authentication or account registration. To enhance registration security and prevent bot registrations, Keycloak integrates with Google reCAPTCHA [65], providing an additional layer of protection against spam and misuse. By adopting Keycloak as the identity manager, there is the capability to delegate authentication to third-party identity providers, utilizing standard protocols such as OAuth 2.0. Keycloak’s Identity Management component facilitates user registration, authentication, and role assignment within the e-VITA platform. All e-VITA components are secured, requiring users to authenticate against the federated identity manager and obtain the necessary authorization. This ensures consistent user identification, associating specific data with individual users during information collection or granting access only to data related to a specific user.

**Figure 15:**
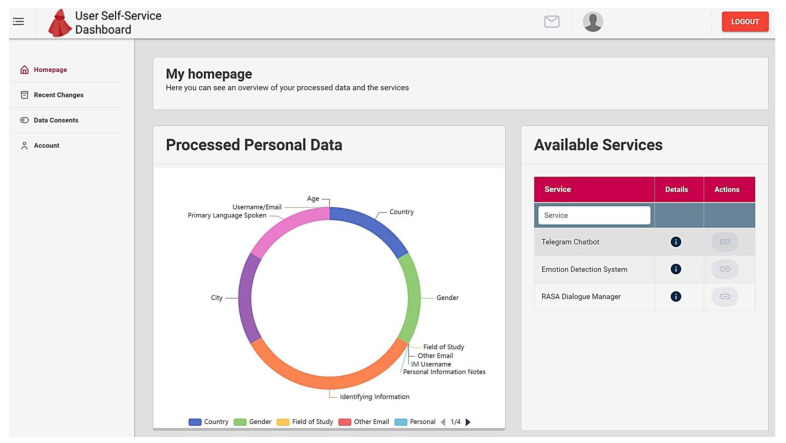
e-VITA Privacy Dashboard.

For effective privacy management and consent, the CaPe ICT suite is integrated, serving as both a data controller and processor. CaPe handles user-centered personal data based on consent in interactions involving data subjects and public and private services. Users can grant and withdraw consent for third parties to access their personal data. The e-VITA Dashboard also includes privacy-focused dashboard interfaces for managing services and consents (Figure 15).

To enable users to provide and manage consents for services managed by CaPe, the e-VITA Dashboard incorporates a dedicated Privacy Dashboard component. A set of services has been defined for managing user consent regarding the sharing of their data with external applications. In the context of this system, the e-VITA platform acts as a data provider, serving as a repository for user data, while external applications that require access to the stored data for analysis are considered service providers (e.g., Chatbots, Emotion Detection System, and RASA Dialogue Manager). Within the Privacy Dashboard, users can explore a list of services, each accompanied by a description outlining its purpose. Users have the option to grant consent for the utilization of personal data to fulfill the specified purposes of each service. Registered services are presented within the Privacy Dashboard, accessible to any registered user. Users can review the available services, modify their consents, or revoke consents previously granted. This approach empowers users to make informed decisions about which personal data they wish to share with external services that rely on specific user information for further analysis. Users also retain the ability to withdraw their consent at a later time. Detailed additional information on the e-VITA platform security and privacy layer can be found in the project deliverable “D6.11 High-fidelity Demonstrator of the AHA Privacy Dashboard” [4].

### 4.5. Visualization and End-User Applications

#### 4.5.1. The e-VITA Dashboard

**Figure 16:**
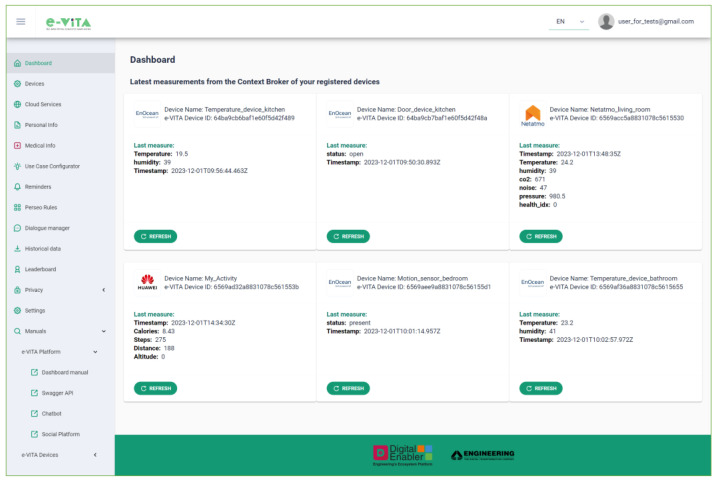
e-VITA Dashboard.

The e-VITA Dashboard serves as the main user interface for accessing the functionalities of the e-VITA platform (Figure 16). Integrated with the Keycloak Identity and Access Management tool, it facilitates user registration, authentication, and authorization with assigned roles such as administrator, end-user, tester, developer, and researcher.

Different roles grant varying levels of access to specific sections of the platform. 

**Figure 17:**
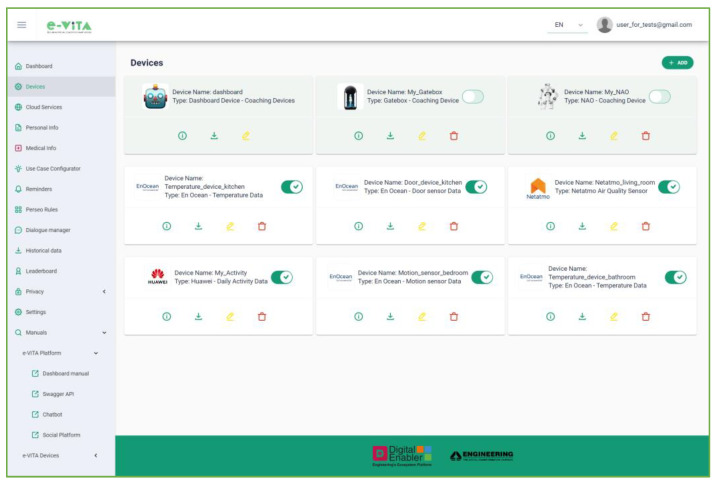
e-VITA Dashboard—devices section.

**Figure 18:**
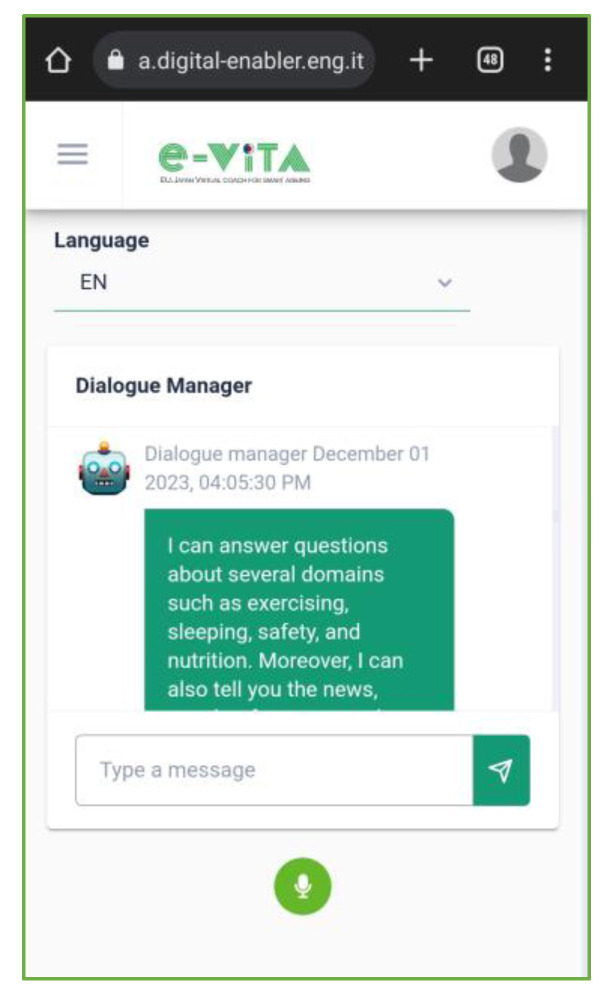
e-VITA Dashboard—dialogue manager section accessed from smartphone.

**Figure 19:**
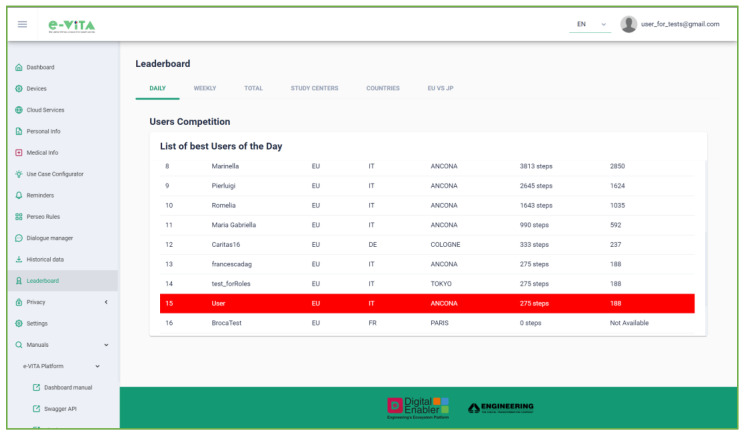
e-VITA Dashboard—leaderboard section.

The dashboard allows users to perform the following actions:

- View the latest measurements from personal devices, communicating with the FIWARE Orion Context Broker;
- Manage registered devices, enabling/disabling and viewing detailed information, downloading measurements, and editing/deleting devices (Figure 17);

- Access external cloud services (e.g., Netatmo, Neu, and Huawei) for devices connected to these platforms in order to obtain the measurements taken;
- View personal and medical information entered by a human coach user supporting older individuals;
- Interact with the Use Cases Configurator, detailed in Section 4.5.2, to input the needs and requirements of the older user and view processing results;
- Configure reminders using the Esper-based Complex Event Processing (CEP) FIWARE Perseo component, which allows the creation of alerts that will be automatically notified to the user via the selected device. Configuration involves selecting a certain number of repetitions, how long before the event the reminder will be sent, and the time interval between alerts;
- Communicate with the Rasa Dialogue Manager via text or audio, changing language and receiving voice responses. This functionality is particularly useful when accessing it directly from a smartphone (Figure 18). The e-VITA platform is a responsive app that can be accessed from desktops, mobiles, tablets, or any other interface, enabling users to have a better experience regardless of the device, screen size, orientation, and browser platform;
- Obtain historical data for analysis, respecting user anonymity;
- View leaderboards on users’ achievements in terms of steps and distance, fostering an active lifestyle (Figure 19). The values in these rankings are updated daily. Specifically, the rankings show data for the current day, weekly data, i.e., for the last seven days, and total data, i.e., the number of steps taken by each user since the start of the trial. An additional ranking compares the users’ data, creating competition between the different study centers (located in Sendai, Tokyo, Natori, Cologne, Siegen, Paris, and Ancona), between different countries (France, Germany, Italy, and Japan), and finally between the two considered continents (Europe and Japan). In the rankings, the position of the current user is highlighted;
- Manage the privacy of personal data through interaction with the CaPe component;
- Access user manuals and documentation for proper platform and component use.

From a technological point of view, the implementation of the dashboard is based on ngx-admin (Akveo LLC, Lakewood, CO, USA, 2022) [66], an open-source responsive dashboard template based on Angular and Nebular that includes some of the most used Angular libraries (e.g., Bootstrap, Fontawesome, and Leaflet). 

#### 4.5.2. Use Cases Configurator

**Figure 20:**
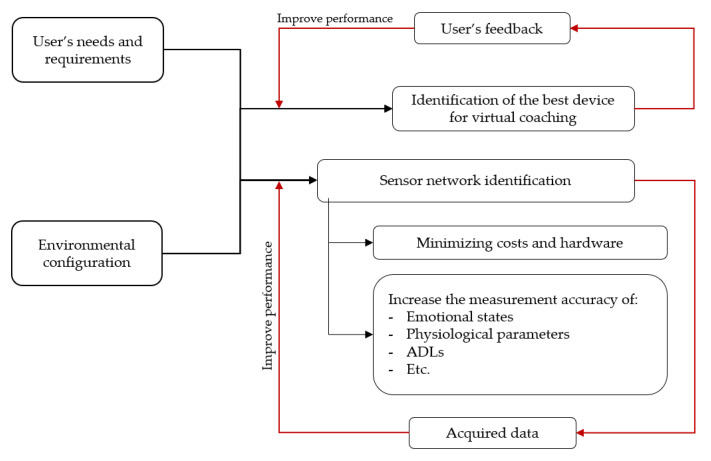
Conceptual diagram of the Use Cases Configurator.

The Use Cases Configurator (UCC) is a software component of the e-VITA platform designed to translate user needs and requirements and environment configuration into technical specifications of the sensing and coaching system [67]. Acting as an interface for technical installers and formal caregivers, the UCC focuses on creating a smart living environment that balances expenses and sensor quantity while maintaining measurement precision (Figure 20). Additionally, it aims to identify the optimal virtual coaching device.

The UCC features a graphical interface that empowers installers with a variety of options to select from. These choices are informed by the user’s information, preferences, and objectives. Inputs for the configurator include user needs, details about the living environment (like house structure and room count), dwelling situation (single resident or multiple residents), preferences for wearable or stationary sensors, privacy settings, and personal particulars (such as gender, age, cultural elements, and religion). After processing this data, the configurator generates the most suitable sensor network configuration for the specific use case. It classifies sensing technologies and coaching devices based on these inputs, resulting in a cohesive and integrated sensor network. This network is adept at discerning user behaviors, physiological conditions, and emotions, as well as selecting an acceptable coaching device. The configurator optimizes the sensor network to avoid user dissatisfaction regarding excessive sensors while minimizing implementation costs and removing irrelevant data to enhance the data analysis system.

Primarily utilized during the initial phase of e-VITA system deployment, the configurator serves as a valuable resource for installers responsible for setting up sensor and coaching device networks within the homes of older users or care institutions. This approach ensures that users receive a customized service tailored precisely to their preferences and requirements.

#### 4.5.3. e-VITA Smartphone App

The e-VITA project includes a smartphone app for end users, serving as a centralized control hub and providing a single point of access to external applications. Users can conveniently access various applications and interventions, including Telegram Chatbots for coaching older adults, through this app.

The e-VITA smartphone app hosts both e-VITA-specific applications, such as a social platform, and external applications, including those managing system settings like the CaPe Privacy Dashboard (explained in Section 4.4). Users can access the control center via the app to conveniently reach the CaPe Privacy Dashboard, allowing them to review permissions granted for specific personal data to specific services, leveraging the authentication capabilities of Keycloak.

The social platform seamlessly integrates local interest groups, fostering connections among individuals of varying age groups. The platform aims to create an environment where senior citizens can share valuable life experiences with younger generations and easily explore local events and volunteer opportunities. Accessing the social platform via the e-VITA app is simple, with users registering or logging in through the app to gain platform access. To register, users must complete a personal information form. For existing registrants, they can simply enter their credentials to access the homepage. Users can hold different roles, including Administrator, Stakeholder, User Administrator, Service Providers (with or without certification), and Primary Users, granting access to fundamental website functionalities. The social platform application is customized to different countries and communities, focusing on local locations and communities surrounding the primary end users.

To enhance the physical activity and well-being of older individuals, the e-VITA mobile app incorporates two distinct Chatbots within the Telegram app. One focuses on exercise-related guidance, while the other provides nutritional assistance.

Leo 2.0, the exercise chatbot, supports an active lifestyle by providing customized exercise videos based on user preferences. It suggests personalized exercise options based on the user’s target body area and fitness goals. In addition, the chatbot keeps a history of exercise video selections made by users. Users can conveniently set reminders and manage their list, with the ability to delete reminders if necessary. Furthermore, the chatbot actively encourages users to provide feedback on its performance and share their opinions, ensuring ongoing improvements and customization to better meet the needs of the users.

Fridolin, the nutritional chatbot, helps users acquire detailed nutritional data on their meals, including macronutrient amounts and calorie content. It provides tailored recommendations aligned with the macronutrient requirements of users, basal metabolic rate (BMR), and daily calorie needs. Users can voluntarily share certain information for personalized suggestions, and Fridolin maintains a record of this data to provide consistent and customized nutritional guidance. Whether users choose to share their data or not, Fridolin remains committed to offering valuable meal recommendations and nutritional insights to support informed dietary decisions.

## 5. Platform Evaluation

### 5.1. Implementation of e-VITA Devices

The Proof-of-Concept study of the e-VITA project has a duration of six months and will end in January 2024. It is conducted in three European test centers (Germany, Italy, and France) and three Japanese test centers, with Ethics Committee approval in the participating countries.

The main purpose of the study is to evaluate participant adherence to the virtual coach system through the analysis of usage frequency and dropout rates after validation of the functioning of the technical platform. Additionally, it aims to assess the improvement of participants’ quality of life. Secondary objectives involve evaluating the usability, user experience, acceptability, and fulfillment of needs related to the e-VITA system. The research also investigates potential changes in health-related areas, including physical activity, cognition, nutrition, loneliness, and health literacy.

A total of 240 older volunteers, each test center enrolling 40 subjects, participated in the study. The tests were performed in the older adults’ homes, where e-VITA devices were installed. The participants were retirees over 65 years old, free from uncontrolled health problems, capable of standing and walking unaided, and possessed the capacity to provide informed consent. They were assigned to an experimental or control group in an alternating, randomized manner based on the order of inclusion. 

For participants in the experimental group, the following devices were assigned in a randomized, alternating manner according to their order of inclusion:

- NAO;
- CelesTE (Europe)/DarumaTO (Japan);
- Gatebox;
- Tablet.

Additionally, all participants in the experimental group were equipped with the following:

- Wearable and home sensors on the e-VITA platform detect physiological parameters, monitor physical activities, and analyze user behavior. The specific sensors made available were contingent on the participants’ residence in either Europe or Japan, as indicated in Table 2. Participants were given the autonomy to decline the use of any sensors they did not wish to incorporate into their setup;
- Smartphone, to enhance their interaction with the virtual coach. The smartphone likely played a role in facilitating communication with the virtual coach and housed a chatbot for insights, suggestions, and stimulation related to healthy aging practices;
- Booklet on active and healthy aging, offering information and activities on well-being.

On the other hand, participants in the control group only received a booklet on active and healthy aging. This random allocation ensures a diverse representation of participants across different devices in the experimental group, allowing for a comprehensive evaluation of the impact of various technologies on the study outcomes. The control group, receiving only a booklet, serves as a baseline for comparison against the interventions provided to the experimental group.

Table 6 shows the different configurations of devices deployed in the homes of older participants in each of the different test center countries, randomly assigned (five participants per configuration).

Participants in the experimental group benefited from a training session before implementing the e-VITA devices. During this session, participants were introduced to the content of the programs offered, covering nutrition, cognition, sleep, socialization, and spiritual life. During this session, they also familiarized themselves with the e-VITA devices and learned how to use them.

The participants of the experimental group were evaluated at T0 (1st month), i.e., at the time of implementation of the e-VITA devices, then at T1 (3rd month) and T2 (6th month) with questionnaires and interviews. Furthermore, regular follow-ups were conducted during the experiment, and human coaches were organized to meet with these participants to assist with any difficulties encountered and to learn about the use of the e-VITA devices. Participants in the control group were evaluated at T0, T1, and T2 with questionnaires.

During the six-month experiment, participants in the experimental group were encouraged to use their assigned devices in a way that reflected their natural behavior outside the context of the experiment. This approach aimed to capture authentic user interactions and experiences throughout the study.

In addition, during the experimental phase, trained human coaches, including students, therapists, or assigned volunteers, engaged with participants bi-weekly. The coaches gathered information on interaction duration, types of interactions, feelings about activities, and socio-economic details. Their role included assisting with device control, addressing difficulties, determining user satisfaction, motivating device use, and providing additional information. Moreover, participants had the option to join videoconference sessions and undergo an intermediate evaluation after three months, conducted by a researcher at their homes. This evaluation involved completing various questionnaires to gather detailed insights into their experiences and perspectives. The multifaceted approach involving human coaches and evaluations aimed to comprehensively understand participant engagement and feedback during the study.

### 5.2. Results

This section provides preliminary results from the data collected in the experimental group at T1 (3rd month).

#### 5.2.1. Target Population for the e-VITA Devices

Participants believed that the system was useful for isolated individuals as it facilitates interaction with the outside world: “*it’s good for people who don’t have many relationships*”. However, participants acknowledged that good cognitive functions were necessary to use the technologies effectively: “*you must have good intellectual functions to be able to use these technologies*”.

#### 5.2.2. Features of the e-VITA Devices

Participants highlighted technical difficulties, especially at the start of the experiment. Challenges included initiating and interacting with the robot: “*I ask it a question and it says: I will think and answer you, but it does not answer me*”. Some participants noted difficulties initiating programs with the mobile phone: “*everything is small on the mobile phone, I can’t see well*” and suggested initiating them on the tablet rather than on a mobile phone. Furthermore, the participants complained about the cumbersome and time-consuming identification steps that sometimes ended in failure: “*you have to put the identifier, then the password, then the date of birth and everything stop because there is a bug*”.

#### 5.2.3. Training Needed by Participants to Use the e-VITA Devices

Participants emphasized the importance of being trained to navigate the interface and use the devices. They expressed satisfaction with the learning session and follow-up by coaches during the five-month intervention. “*The coaches were very friendly and available, giving advice on using the programme and the technology*”. However, they desired an additional day of rehearsal. “*It would have been nice to have a second day of training at Broca Hospital (French test center). We could have the training on the first day, we could try to practice for a week, then we could ask questions to the trainer on the second day*”.

#### 5.2.4. Potential Benefit on Isolation and Feeling of Loneliness

Participants believed that the device could reduce isolation and loneliness by creating a small community of e-VITA users. “*During the first day of training at Broca Hospital, there was a good atmosphere, we had lunch, we talked and we kept in touch. A small community has formed which is very responsive. We discussed our difficulties with technologies and gave each other advice. Then we went on outings together (restaurant, cinema, exhibitions)*”. A second advantage was to benefit from a wide program of activities that allowed them to have fun and learn about many subjects: “*we can do so many activities. It’s not possible to be bored*”. A third advantage was increased visits and requests from people around them (family, friends, and neighbors) due to the interest generated by the e-VITA devices, especially the robots. “*My grandchildren come to see me a lot more. They are fascinated by the robot*”. “*My neighbour is very interested in all the programmes. She often comes to see me and says what do they think of this or that in your nutritional programme?*”

#### 5.2.5. Potential Benefit on Physical Health

Participants also believed the device could be helpful for their physical health. “*I do physical exercises alone at home. This is very good because I didn’t have a place to join a gymnastics group in my neighbourhood. I also connect to the physiotherapists’ website online. Regarding sleep, I have read some very interesting articles. The programme also offers other readings if you want to know more. The cognitive stimulation programme is a bit brief. I hope it will be enriched. Furthermore, I also really appreciated the nutrition programme which is very complete. I learned a lot of things; I think I’m doing something for my health*”. The sensors were also interesting for people. “*I really appreciated the sensor that studies the ambient air at home. All you have to do is open the window for 10 min and you can see a big change in the CO_2_ content. Until now, I had never taken care of it. However, it is important*”.

#### 5.2.6. Participants’ Perspectives on e-VITA Devices

Participants who experienced fewer technical difficulties showed good acceptance of the technology. “*At first, I was scared by the technology, but then you get used to it*”. They would like to continue experimenting with and using e-VITA devices in the future. “*I will miss the presence of the robot even if it has a lot of difficulty responding to me appropriately. I would like to continue to benefit from the programme after the end of the experiment*”.

#### 5.2.7. Ethical Aspects Associated with e-VITA Devices

No ethical concerns were raised by the participants. They did not feel any loss of autonomy related to the presence of virtual coaching: “*I cannot feel controlled or directed because I watch and do what I want and when I want. There is no constraint*”. They did not feel stigmatized. “*I didn’t feel stigmatized. My children were very impressed that I could manage all this technology. It was an honor for me to participate in the project. My friends were jealous*”.

To summarize these preliminary data, participants valued the sense of community facilitated by the e-VITA coaching system. They appreciated specific technologies, in particular the connected smartwatch and the robot that had a very playful nature, and the applications that provided them with information to better monitor their health (such as the Telegram Chatbots). They expressed interest in continuing to use these technologies and provided constructive feedback on the user interface’s ease of use. “*The programme is very good, the problem is not to get lost in the technology due to technical problems, that would be a shame*”.

## 6. Conclusions

The paper offers an in-depth exploration of the e-VITA Platform Architecture, shedding light on its software components, associated technologies, and data flow mechanisms. Serving as the foundation for the coaching system, the e-VITA platform provides crucial functionalities that foster seamless communication and integration among diverse devices. It ensures the harmonious management of collected data, delivering a range of data processing and analytical capabilities vital for the success of coaching interventions. Furthermore, this architecture prioritizes security and privacy, aligning with legal frameworks such as GDPR and APPI. 

The architecture outlined in this paper refers to the current implemented and deployed version of the e-VITA platform, specifically designed to support the experimentation phase of the coaching system [68]. The platform’s functionalities are currently under evaluation in the Proof-of-Concept study, ongoing in Italy, France, Germany, and Japan, involving individuals aged 65 and older who are retired and living independently at home. Preliminary feedback from the experimentation highlights that older participants value community involvement, appreciate interactive technologies, particularly smartwatches and playful robots, find health monitoring apps useful, and emphasize the importance of improving overall usability, especially the interface. While the current architecture is stable and mature in terms of the provided capabilities, it remains flexible and adaptable. Beyond the project’s conclusion, updates and enhancements can be applied to address potential issues and cater to emerging needs identified during the experimentation phase. This adaptability underscores the commitment to delivering effective and responsive solutions in the ever-evolving landscape of smart aging and virtual coaching.

## Figures and Tables

**Table 1:** Comparison of projects and their contributions.

Aspect	Projects											
	WellCo	HoloBalance	EMPATHIC	Council of Coaches	NESTORE	CAPTAIN	vCare	SAAM	NEDO 2.0	METI/AMED	CARESSES	e-VITA
Behavior Change and Intervention	✓		✓	✓	✓	✓						✓
Continuous Monitoring	✓		✓	✓	✓	✓	✓					✓
Affective-Aware Virtual Coach	✓		✓	✓		✓						✓
Interdisciplinary Team	✓		✓	✓		✓	✓					✓
Balance Disorders Coaching		✓										
Real-time Emotional State Extraction			✓			✓						✓
Open Dialogue for Personalized Plans				✓			✓					✓
Multi-Party Interaction				✓								
Wearable Integration					✓							✓
Augmented Reality Integration		✓				✓	✓				✓	
Rehabilitation Guidance							✓					
Ambient Sensing for Coaching								✓				✓
Cultural Capability in Robotics			✓								✓	✓
Chatbot Interface with Multilingual Support			✓								✓	✓
Social Robots for Reminders and Social Interaction									✓	✓	✓	✓

**Table 2:** e-VITA platform sensing devices.

Company	Product	Description	Measured Data
Netatmo	Smart Indoor Air Quality Monitor 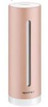	Smart device for measuring indoor environmental parameters (for European use).	Temperature (°C), humidity (%), noise (dB), CO_2_ (ppm).
Delta Dore	DMB Tyxal+ 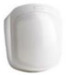	Device that monitors the home environment and user behavior (for European use).	ON/OFF status upon detection of user movement.
Delta Dore	DO Tyxal+ 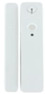	Device that monitors the home environment and user behavior(for European use).	ON/OFF status upon detection of door opening and closing.
EnOcean	ETB-RHT 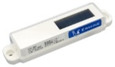	Smart device for measuring indoor environmental parameters (for Japanese use).	Temperature (°C), humidity (%).
EnOcean	ETC-PIR 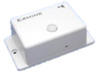	Device that monitors the home environment and user behavior (for Japanese use).	ON/OFF status upon detection of user movement.
EnOcean	ETB-OCS 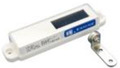	Device that monitors the home environment and user behavior (for Japanese use).	Sensor that provides ON/OFF status upon detection of door opening and closing.
OURA	Ring 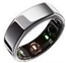	Smart ring that tracks the user’s sleep patterns and physiological parameters.	HRV (ms), HR (bpm), respiratory rate (rpm), burned calories, inactivity time (h), steps, sleep timing (h).
Huawei	Band 7 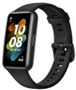	Wristband that monitors the user’s physiological parameters.	HRV (ms), HR (bpm), SpO2 (%), activity level (index), body temperature (°C), burned calories, sleep duration (h), steps, sleep quality (index).
NeU Corporation	XB-01 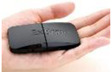	Wearable smart device that measures the user’s brain activity while worn on the forehead.	Brain activity (index).
-	uSkin pillow 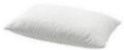	Smart pillow developed in the project that monitors sleep parameters via embedded force sensors.	Sleep quality (index), sleep duration (h).

**Table 3:** Dataset distribution of individual language categories.

Language	Number of Samples
English	11,970
Italian	9409
Japanese	4163
German	454

**Table 4:** Dataset distribution of emotion labels.

Emotion	Number of Samples
Anger	3978
Disgust	4444
Fear	3593
Happiness	4412
Neutral	3158
Sadness	4188
Surprise	2223

**Table 5:** Classification report for SER.

Emotion	Precision	Recall	F1-Score	Support
Anger	0.71	0.67	0.69	600
Disgust	0.58	0.66	0.62	668
Fear	0.58	0.56	0.58	541
Neutral	0.59	0.56	0.57	663
Happiness	0.61	0.56	0.58	475
Sadness	0.62	0.68	0.64	631
Surprise	0.68	0.70	0.70	335

**Table 6:** Configuration of e-VITA system devices installed in the homes of older participants for each test country. Each configuration was randomly assigned to 5 participants per test center.

Country	Coaching Device	Wearable Device	Home-Based Device	Support Device
Italy, France, Germany	NAO	Huawei Band 7	Delta Dore DMB TYXAL+ Delta Dore DO TYXAL+ Netatmo	Smartphone
	Gatebox	Huawei Band 7	Delta Dore DMB TYXAL+ Delta Dore DO TYXAL+ Netatmo	Smartphone
	CelesTE	Huawei Band 7	Delta Dore DMB TYXAL+ Delta Dore DO TYXAL+ Netatmo	Smartphone
	Tablet	Huawei Band 7 NeU XB-01	Delta Dore DMB TYXAL+ Delta Dore DO TYXAL+ Netatmo	-
Japan	NAO	Huawei Band 7	EnOcean ETC-PIR EnOcean ETB-OCS EnOcean ETB-RHT	Smartphone
	Gatebox	Huawei Band 7	EnOcean ETC-PIR EnOcean ETB-OCS EnOcean ETB-RHT	Smartphone
	DarumaTO	Huawei Band 7	EnOcean ETC-PIR EnOcean ETB-OCS EnOcean ETB-RHT	Smartphone
	Tablet	Huawei Band 7 NeU XB-01	EnOcean ETC-PIR EnOcean ETB-OCS EnOcean ETB-RHT	-

## Data Availability

No new data were created or analyzed in this study. Data sharing is not applicable to this article.
